# Jojoba Oil: An Updated Comprehensive Review on Chemistry, Pharmaceutical Uses, and Toxicity

**DOI:** 10.3390/polym13111711

**Published:** 2021-05-24

**Authors:** Heba A. Gad, Autumn Roberts, Samirah H. Hamzi, Haidy A. Gad, Ilham Touiss, Ahmed E. Altyar, Osama A. Kensara, Mohamed L. Ashour

**Affiliations:** 1 Department of Pharmaceutics and Industrial Pharmacy, Faculty of Pharmacy, Ain Shams University, Cairo 11566, Egypt; h.gad@pharma.asu.edu.eg; 2 Independent Researcher, La Route de la Haule, St. Peter, Jersey JE3 7BA, UK; autumnggroberts@yahoo.co.uk; 3 Department of Pharmaceutical Sciences, Pharmacy Program, Batterjee Medical College, P.O. Box 6231, Jeddah 21442, Saudi Arabia; 120073.sameerah@bmc.edu.sa; 4 Department of Pharmacognosy, Faculty of Pharmacy, Ain Shams University, Cairo 11566, Egypt; haidygad@pharma.asu.edu.eg; 5 Laboratory of Bioresources, Biotechnologies, Ethnopharmacology, and Health, Faculty of Sciences, University Mohamed I, Oujda 60000, Morocco; i.touiss@ump.ac.ma; 6 Department of Pharmacy Practice, Faculty of Pharmacy, King Abdulaziz University, P.O. Box 80260, Jeddah 21589, Saudi Arabia; aealtyar@kau.edu.sa; 7 Department of Clinical Nutrition, Faculty of Applied Medical Sciences, Umm Al-Qura University, P.O. Box 7067, Makkah 21955, Saudi Arabia; oakensara@uqu.edu.sa

**Keywords:** jojoba, *Simmondsia*, chemistry, liquid wax, biology, toxicity, pharmaceutical/industrial uses

## Abstract

Jojoba is a widely used medicinal plant that is cultivated worldwide. Its seeds and oil have a long history of use in folklore to treat various ailments, such as skin and scalp disorders, superficial wounds, sore throat, obesity, and cancer; for improvement of liver functions, enhancement of immunity, and promotion of hair growth. Extensive studies on Jojoba oil showed a wide range of pharmacological applications, including antioxidant, anti-acne and antipsoriasis, anti-inflammatory, antifungal, antipyretic, analgesic, antimicrobial, and anti-hyperglycemia activities. In addition, Jojoba oil is widely used in the pharmaceutical industry, especially in cosmetics for topical, transdermal, and parenteral preparations. Jojoba oil also holds value in the industry as an anti-rodent, insecticides, lubricant, surfactant, and a source for the production of bioenergy. Jojoba oil is considered among the top-ranked oils due to its wax, which constitutes about 98% (mainly wax esters, few free fatty acids, alcohols, and hydrocarbons). In addition, sterols and vitamins with few triglyceride esters, flavonoids, phenolic and cyanogenic compounds are also present. The present review represents an updated literature survey about the chemical composition of jojoba oil, its physical properties, pharmacological activities, pharmaceutical and industrial applications, and toxicity.

## 1. Introduction

The plant kingdom continues to hold considerable importance in our daily life. In addition to supplying humanity with food, it is considered as a potential source of thousands of novel materials such as fragrances, flavoring agents, dyes, fibers, beverages, building materials, heavy metal chelators, and many useful compounds of great therapeutic value. 

Early studies of plants helped humankind make use of local flora for healing ailments. These studies have continued until now to seek out new agents for the treatment of various diseases. Recent investigations regarding plants with centuries of use in folk medicine have generated a great deal of information about the biologically active chemical components responsible for many claimed medicinal effects. As a result of thorough research involving the isolation and structure characterization techniques, many lead compounds, and prototypes from natural products have assumed reputable roles in medicine. Despite the huge number of synthetic and semisynthetic drugs, the most valuable medicinal agents still in use are obtained from medicinal plants.

There is high consumption of natural resources due to the increasing population. The resultant demand for green energy amidst fossil fuel shortages has rekindled interest in Jojoba oil (*Simmondsia chinensis* (Link) Schneider). Jojoba oil is the only unsaturated liquid wax readily extractable in large quantities from plant sources (≈52% of the total seed weight), which shows a high structure similarity with the sperm whale oil. This similarity has increased the interest in Jojoba oil as a replacement for sperm whale oil (spermaceti wax) since the 1970s [1].

*Simmondsia chinensis* (Link) Schneider is native to North and Central American deserts but cultivated worldwide in Chile, Egypt, and Argentina [2]. Jojoba was widely used by Native Americans in the Sonora desert (California) as a foodstuff in the form of cooked fruits and in oil form as a therapeutic for multiple ailments: cancer therapy, liver and kidney disorders, obesity, parturition, sore throat, superficial wound healing, warts, psoriasis, acne, sunburn, and treatment of poison ivy exposure [3,4,5,6]. Jojoba oil is widely used in the pharmaceutical industry, especially in cosmetics, to restore the ordinary health of hair and skin. The leaf extract, combined with extracts from other plants, also acts as anti-inflammatory agents to treat sensitive skin stress [7]. Jojoba cosmetic products currently on the market include the following: bath oil, body oil, cleansing creams, cleansing pads, cleansing scrubs, nourishing facial cream, facial oil, hair conditioner, hair oil, makeup remover, and shaving cream [8,9,10,11]. 

In addition, the oil has many industrial applications that include an extreme temperature/extreme pressure lubricant in the form of sulfurized oil, which can bear high temperature and pressure without changing its viscosity [6,12]. Other industrial uses include the extraction and separation of isotopes such as Uranium (VI), Thorium (IV), and Plutonium (IV); in the leather industry as a fat liquor with good tanning properties [5]; as a surfactant, fire retardant, lamp oil, candle wax, polishes [13], and antifoaming agents in isolation of penicillin and tetracycline [9].

An updated and in-depth review about jojoba oil chemistry, its pharmaceutical and industrial uses, and toxicity was conducted and is presented in this work to supplement the lack of comprehensive reviews covering the plant since the 1990s. The keywords jojoba, *Simmondsia*, chemistry, pharmaceutical preparations, emulgels, nanoparticles, toxicity, and biological activity were used in many combinations to search Scifinder^®^, PubMed^®^, Web of Science ^®^ starting 1990 until 2021. English language was used as the only filter.

## 2. Common Names and Botanical Characteristics

The word jojoba, pronounced “ho-ho-ba”, is a distortion of the native Papago Indian word “howhowi”. Jojoba is known by many other names such as bucknut, coffee nut, goatnut, pignut, nutpush, goatberry, sheepnut, and lemon leaf [14]. The seeds of the jojoba plant are dark brown, akin to large coffee beans.

**Figure 1:**
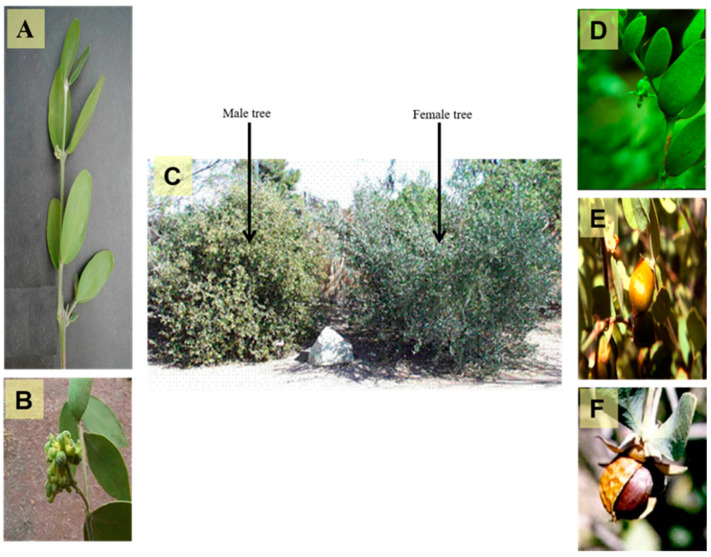
A photograph showing different organs of *Simmondsia chinensis*, (**A**) Branch of the plant (X 0.8), (**B**) Male flowers (X 1.0), (**C**) Old male and female trees (X 0.02), (**D**) Female flower (X 0.5), (**E**) Ripe fruit (X 0.5) and (**F**) Seed (X 0.8) (Photographer Eng. Nabil Elmougi, the jojoba farms of The Egyptian Natural Oil Company, Ismailia Desert Road, Egypt).

Plants of the order Euphorbiales are usually herbs, shrubs, or sometimes trees [15]. They are widely distributed globally, especially in temperate, subtropical, and tropical regions [16]. They have frequently unisexual (rarely bisexual) hypogynous flowers, which are generally regular with a single whorl of a green perianth. The stamens are equal in number to perianth leaves or numerous. The pistil is composed of three carpels forming a trilocular ovary, with each chamber containing one or two anatropous, pendulous ovules in the inner angle with ventral or dorsal raphe [17]. Simmondsiaceae is a small family of one genus, *Simmondsia*, which is abundant in Southern Arizona, Sonora, and Baja California. Plants that belong to “Simmondsiaceae” are mostly woody branched shrubs that reach 2–4 m in height [4,5,6]. A photograph of male and female trees, flowers, and seeds of *Simmondsia chinensis* are displayed in Figure 1.

## 3. Chemical Constituents

Jojoba oil is composed of almost 98% pure waxes (mainly wax esters, few free fatty acids, alcohols, and hydrocarbons), sterols, and vitamins with few triglyceride esters, so it is widely known as liquid wax rather than oil or fat [18].

### 3.1. Jojoba Wax

Investigation of the different organs of the jojoba plant for the presence of the wax revealed that the seeds contain most of the wax content in the plant (almost 50–52% of the seed weight) [5]. Jojoba wax is composed mainly of esters and, to a lesser extent, free acids, free alcohols, and hydrocarbons [4]. Esters are composed by the association of long straight-chain fatty acids with long straight-chain or higher molecular weight monohydric alcohols, C20 and C22; both the acids and alcohols are cis-monounsaturated at the (ω-9) position. Small triglyceride esters are also present [19,20,21,22].

#### 3.1.1. Wax Esters

The main components of the wax esters that have been isolated and previously identified are docosenyl eicosenoate “erucyl jojobenoate” (1), eicosenyl eicosenoate “jojobenyl jojobenoate” (2), eicosenyl docosenoate “jojobenyl erucate” (3), docosenyl docosenoate (4), eicosenyl oleate (5), and docosenyl oleate (6) (Table 1) [23]. Many other wax esters and free fatty alcohols and acids components are present in small quantities [19,24]. 

It was initially thought that jojoba wax esters were made up of random combinations of alcohols and acids until Miwa conducted a study on these combinations [13]. He showed a significant difference between the observed results and those calculated by a random association of acids and alcohols. For instance, it was observed that (acid/alcohol, % experimental (% random)): (C20:1/C’20:1, 28.0% (31.8%)), (C20:1/C’22:1, 10.3% (5.7%)), (C22:1/C’20:1, 41.4% (32.0%)), (C22:1/C’22:1, 1.9% (5.7%)) indicating that eicosenyl docosenoate ester is preferably biosynthesized by the association of eicosenoic acid and docosenol. These combinations demonstrate that plants favor specific associations, which correspond to their genome. From an analytical point of view, this observation constitutes a valuable tool for detecting adulterated oil and good discrimination between natural Jojoba wax and its synthetic substitutes. In the latter case, associations between fatty acids and alcohols are governed by thermodynamic rules, and random results would be observed. 

#### 3.1.2. Free Fatty Acids and Alcohols

It was reported that the natural oil contains small quantities of free fatty acids (0.96%) and free alcohols (1.11%), as seen in (Table 2) [13].

### 3.2. Sterols

**Figure 2:**
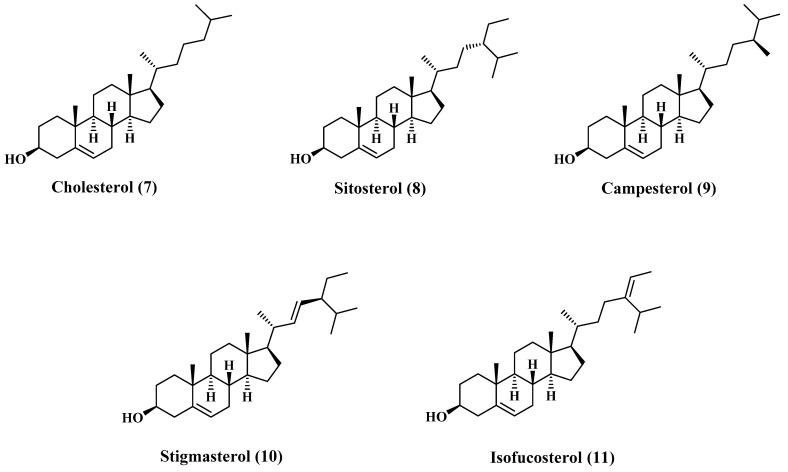
Structures of major sterols content of jojoba oil.

There are many reports concerning the sterol content of jojoba oil [5,19]. The major content of the sterols fraction is cholesterol (7), β-Sitosterol (8), campesterol (9), stigmasterol (10), and isofucosterol (11). Most of these sterols are sketched in Figure 2, and the composition is tabulated in Table 3 [22]. 

### 3.3. Flavonoids, Phenolic, and Cyanogenic Compounds

Although phenolic compounds are the most common secondary metabolites distributed in nature, they are only present in small quantities in jojoba, as reported in Table 4 [25]. Ten flavonoids have been identified as quercetin (12), quercetin 3′methyl ether (isorhamnetin), quercetin 3-methyl ether (14), quercetin 3,3′-dimethyl ether (15), isorhamnetin 3-*O*-glucoside (16), quercetin 3-O-glucoside (17), typhaneoside (18), isorhamnetin 3-O-rutinoside (19), quercetin 3-*O*-rutinoside (20). Some lignans are also present: (+)-lyoniresinol 4,4′-bis-*O*-β-d-glucopyranoside (21), salvadoraside (22), and eleutheroside E (23) [26]. Simmondisin (24), simmonosides A (25), simmonosides B (26), and 4, 5-dimethyl-4-*O*-alpha-d-glucopyranosylsimmondsin (27) are the main cyanogenic glycosides [20,21,27]. 

### 3.4. Fat-Soluble Vitamins

Vitamin D and its derivatives *viz.* α, γ, and δ tocopherol were isolated and quantitatively estimated in the oil where γ-tocopherol makes up approximately 79% of these compounds. Other fat-soluble vitamins such as vitamin A are also found [22].

## 4. Physical Characters of the Oil

Crude jojoba oil obtained directly by either cold expression or solvent extraction of the seeds, without any modifications, yields an oil with a golden or light yellow color. It has a pleasant, slightly nutty taste [6]. The thermal and oxidative stabilities of the oil are high; therefore, the oil shows high resistance toward rancidity due to the presence of natural antioxidants (α, γ, and δ tocopherol) [6]. Refined or bleached oil, obtained by passing the natural oil over activated charcoal and treating with caustic alkali substances, is nearly white with low oxidative stability due to subsequent removal of the antioxidants. The thermal stability of both natural and bleached forms is high, which is indicated by a high flash point reaching 295 °C [28]. The oil’s viscosity favors using the oil and/or its derivatives as an extreme temperature/pressure lubricant [6,12]. Jojoba oil is soluble in common solvents such as benzene and chloroform. However, it is essentially immiscible in methanol. The solubility of jojoba in different organic solvents (Table 5) [29] and other physical properties (Table 6) are listed in many previous works [4,5,6,13,29,30,31].

## 5. Chemical Properties of the Oil

Jojoba molecules contain two double bonds at ω-9 positions in both alcohol and acid sides, which are separated by an ester bond. While in typical plant oils, double bonds are usually close to each other; in jojoba molecules, they are far apart and uneven from the center. These three active sites have been proven to be the source of many intermediates and final products with different physical and chemical characters. These derivatives are described with particular reference to the reaction that leads to the formation of a wide potential of industrial and pharmaceutical applications: the production of semisoft waxes by geometrical isomerization in the manufacture of suppository bases, production of hard waxes by hydrogenation in the manufacture of candles, production of additives by sulfurization for high-pressure/high-temperature lubricants, production of selective exectrants for the nuclear industry by phosphonation of chemically bonded jojoba oil [5,32,33].

These chemical modifications provide a wide range of polymers with diverse properties that could serve as good candidates in industrial application, especially those related to the polyhydroxyurethanes polymers that will be discussed in detail in Section 8.1.

Shani continued his basic research on the possible reaction schemes at the double bond. All-trans jojoba oil was prepared by the straight chemical route, involving the anti-addition of bromine or chlorine to the double bonds followed by displacement and elimination of the halogens with Na I. All-*trans* jojoba was obtained at a yield greater than 75% and had a melting point of 52–54 °C. Similar to those obtained from natural liquid oil, a series of products were prepared from the semisolid all-*trans* jojoba under the same conditions used for the natural liquid oil [34,35]. 

Thorough investigations of the all-*trans* derivatives and their physical and chemical characteristics revealed that the all-*trans* derivatives of jojoba oil had essentially the same melting points as those of the *cis* configuration. Based on these observations, Shani concluded that the polar groups played a more significant role than the geometrical configuration of the double bonds in determining the strength of the packing of the molecules in the solid phase.

### 5.1. Cis/Trans Isomerization

Unsaturated fatty materials can be converted into solid materials by geometrical isomerization of the double bonds. The *trans* isomer of jojoba is thermodynamically more stable than the *cis* form. Moreover, it has a substantially higher melting point, and its soaps have superior wetting and detergency properties. The reaction has never had any commercial significance, which is most likely because the same results can be achieved by partial hydrogenation with the additional advantages of higher oxidation stability. This phenomenon could expand the uses of this isomerized material, especially as a suppository base in the pharmaceutical industry due to its natural creamy appearance coupled with a melting point close to human body temperature [6]. 

Wisniak and Alfandary were the first to report on the geometrical isomerization of jojoba oil with selenium and NO_2_ catalysts under a wide range of conditions. Melting points of the resultant product varied between 36 and 42 °C. Proper adjustment of the operating conditions could, if necessary, allow the preparation of a material with a melting point close to average human body temperature [36]. Later, Galun and coworkers described the thermal and photosensitized isomerization of jojoba oil. The absorption of light at wavelengths of 366 nm or more via the allowance of sensitizers enables the acquisition of the isomerized form of jojoba oil. However, this is dependent on the fact that the *cis* isomer can transform into the trans form if heated to a temperature sufficiently high and that the double bonds present in jojoba oil absorb light of wavelengths below 200 nm as a result [37,38].

### 5.2. Hydrogenation

Hydrogenation is a standard technique for improving the properties of vegetable and animal oils. In addition, it increases the softening and melting points of the fats and improves their color, odor, and stability. The reaction involves the chemical addition of hydrogen to the unsaturated carbon-to-carbon double bonds in fatty alcohol or the fatty acid molecule. This addition occurs by mixing the heated oil and hydrogen in the presence of nickel as a catalyst, at pressures in the order of 0–120 psi, and in hydrogenation conditions widely used in industrial purposes [5]. Total hydrogenation of the oil produces highly lustrous, pearly white crystalline laminae that are very hard. Solid wax has been suggested as a potential ingredient in polish waxes, carbon paper, waxing of fruit, and candles component [5]. 

In 1959, a comparison was made by Knoepfler et al. between the hydrogenation characteristics of jojoba oil obtained by extracting the oil with solvents and those obtained by the cold-hydraulic pressing of the jojoba seeds. The results revealed no significant difference between both hydrogenated forms, except in the melting point. Those prepared from cold-hydraulic pressing have a melting point of 67–68 °C, while those prepared from oil extracted by solvents have a higher melting point of 74–76 °C [32]. Wisniak and Holin have studied the hydrogenation of jojoba oil under a wide range of operating conditions and reaction kinetics in the preparation of different types of solid waxes and compared the characters of the hydrogenated jojoba oil with Beeswax and Carnauba wax. It was observed that jojoba oil is substantially better than Beeswax and relatively equal with Carnauba wax regarding hydrogenation [39]. Simpson and Miwa have done an in-depth X-ray diffraction study of hydrogenated jojoba oil to determine fatty acid and alcohol chain conformation, unit cell, and angle of tilt of the chains [40].

### 5.3. Halogenation

Halogenated fatty materials find extensive uses in preparing quaternary compounds, anti-rotting, flame proofing, and fungicide additives. In addition, brominated vegetable oils have long been used as weighting oil in carbonated beverages [41]. In 1979, Wisniak and Alfandary conducted an extensive study of the chlorination and bromination of jojoba oil. Their main objective was to determine the kinetics of the reaction and evaluate the influence of the operating variables. The experimental results indicated that in the dark and the temperature range used (−15 to +5 °C), a direct addition to the double bond with essentially no substitution occurred. The rate of halogenation decreased with the increase in temperature [42].

### 5.4. Sulfurization and Sulfur Halogenation

The sulfurization of fatty material with sulfur or other reagents containing sulfur and halogen yields various products with different physical and chemical properties. In general, when sulfur content is low (<5%), the products will be liquid and be used as additives. Increasing the sulfur content will increase the viscosity until a rubber-like mass is obtained.

In 1975, Gisser et al. conducted a deep study about the mechanical properties of sulfurized jojoba oil and sulfurized sperm oil. The results obtained revealed that there is no difference between both oils [5]. Furthermore, sulfurized jojoba oil has additional advantageous properties regarding its appearance and high viscosity; the same result was obtained by Miwa et al. [13].

### 5.5. Phosphonation

Dialkyl alkylphosphantes are stable organic phosphorus esters possessing unique properties and offer considerable potential for commercial exploitation. Thus, they have been recommended for use in many applications. As plasticizers, they hold great potential due to their superior stability and other unique characteristics compared to organic phosphates. They have been suggested as synthetic lubricants, additives to improve the extreme pressure properties of lubricants, functional fluids, oil, or fuel additives; pour-point depressants, pesticides, synergists, or carriers for pesticides and fertilizers, intermediates for the synthesis of corrosion inhibitors, and metal extractants. In general, several dialkyl alkylphosphantes are useful as flame-retardants, softeners, textile treating agents, and heat transfer media [5]. Wisniak has reported preliminary experimental data on the phosphonation of jojoba oil with different dialkylphosphites, using tert-butyl perbenzoate as a radical generator. The average ester chain in jojoba oil contains two double bonds so that the final product may contain up to two atoms of phosphorus per chain [33,42].

### 5.6. Oxidation, Epioxidation, and Ozonolysis

Jojoba oil shows good thermal stability up to a relatively high temperature. Generally, the cosmetic formulations containing jojoba oil have superior stability toward oxidation than other lipids used for this purpose. A comparative study of the relative oxidation stability of jojoba oil, sperm whale oil, carnauba wax esters, Limnanthes douglassi wax esters, and behenyl arachidate revealed that jojoba oil has high oxidative stability comparing all other oils [43]. Kampf conducted an in-depth study of the accelerated oxidation of crude jojoba oil and bleached and stripped oils. He found that crude jojoba oil contains natural antioxidants that counted for the high oxidative stability of the natural oil. The removal of these antioxidants through bleaching or stripping of the oil leads to a sharp decline in the oxidative stability of the products [5]. Epoxides of unsaturated glycerides and simple fatty acid esters are currently used as plasticizers and stabilizers for polyvinyl chloride plastics [5]. 

Ozonolysis is an important technique for studying the structure of unsaturated compounds such as those present in jojoba oil. Ozonides in general, but particularly jojoba ozonides, are viable intermediates for many synthetic paths. Zabicky previously used ozone as a reagent to attain intermediates to synthesize different derivatives that are widely used for industrial purposes [5].

## 6. Biological Activity

**Figure 3:**
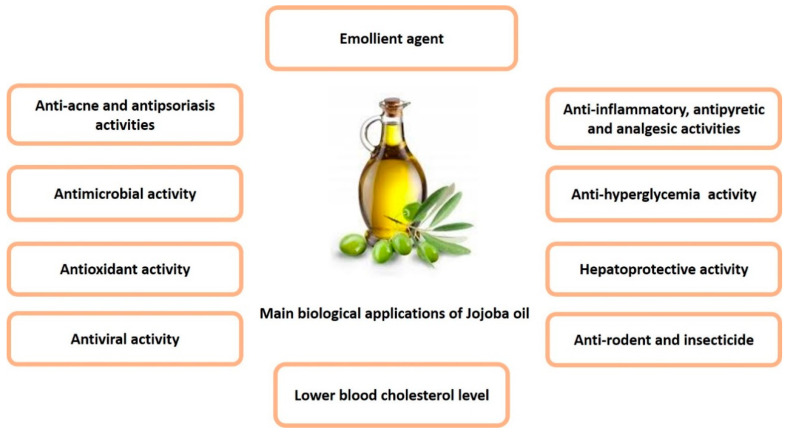
Summary of the main biological activities of jojoba oil.

Extensive biological and pharmacological investigations, based on the uses of jojoba oil in folk medicine, revealed that jojoba oil and its derivatives exhibit vast biological activities in different pharmaceutical forms, whether used topically or internally. These activities can be attributed to the unique chemical composition of the wax esters [4]. Most of the relevant activities were grouped in Figure 3.

### 6.1. Traditional and Folk Medicinal Uses

Jojoba has a rich ethnobotanical history due to its wide use by natives of the arid southwestern deserts of the USA and northwest of Mexico. Jesuit priests in this area recorded tribal uses of jojoba for many skin and scalp disorders. It was first reported in 1789 by the Mexican historian Francisco J. Clavijero that the Amerindians of Baja California highly prized the fruit for food and the oil as a medication [10,33]. “Two to three jojoba seeds taken in the morning are said to be good for the stomach. Seeds when ground and mixed with chocolate facilitate parturition for women. Toasted and ground seeds are found to be specific against sores that erupt on the face. The unguent oil stops chills and if eaten in certain quantity gradually eliminates them”. He was also the first to describe how the Indians use the nuts to treat the wounds, where jojoba nuts were put in hot ashes until the oil starts oozing. “They were then ground on rocks, with the resultant salve-like substance applied to the wound. This salve is claimed to cure cuts, scratches and sores rapidly” [5].

Other early mentioned medical uses include curing the suppression of urine, helping in weight loss, improvement of liver functions, elevating body immunity, remedy for cancer, and promotion of growth of hair [5].

### 6.2. Pharmacological Uses

#### 6.2.1. Emollient Agent

Skin surface-softening effects are represented by an increase in the extensibility or suppleness of the surface. These changes contribute to the overall softness of the skin and make it possible to accommodate stretching and movement without cracks and tears, perceived as scaliness, developing on the surface. That surface suppleness changes rapidly in response to the application of water or known emollients [6,8,10,11,44]. Jojoba oil in single-phase and emulsion systems shows an excellent lubricity without the oily, greasy feel of other lipids, especially lanolin and petrolatum [45]. It can also contribute to superior transpirational water control in the skin, thus reducing evaporation without blocking the passage of gases and water vapor. This character is due to its high molecular weight and low viscosity, and structural similarity with skin sebum, leading to a smoothing effect on dry skins and the inhibition of excess flaking of epidermal cells [5,33]. Skin indentation tests showed that the oil enhanced skin elasticity, similar to the effect of lanolin. Jojoba oil also showed a keratoplastic effect and seemed to restore the skin’s natural shine [5]. 

Many studies have been carried out to evaluate the penetration rates, slip, and occlusive of various emollients, including jojoba oil and fully hydrogenated jojoba oil in many pharmaceutical skin care products. It was found that the derivatives of jojoba oil have excellent lubricity characteristics. It was also demonstrated that hydrogenated jojoba oil has a faster penetration rate and good occlusive properties. Thus, it is recommended to use jojoba oil alone or with other natural oils to maintain the natural appearance of the skin and the safety of that derivative as an emollient in the cosmetic formulation [7,10].

Christensen et al. in 1988 examined jojoba oil, both hydrogenated and ozonized derivatives, for their emollient activity. He found that a marked increase in skin surface suppleness appeared after 5 min which persisted for many hours, implying a potential use in dry skin products. In addition, ozonized jojoba derivatives make the skin surface water repellent and resistant to the stiffening effect encountered after washing with soap and water [6,10].

#### 6.2.2. Anti-Acne and Antipsoriasis Activities

The historic use of jojoba oil by Mexican Indians to treat sores has recently highlighted its potential in treating acne and psoriasis. Miwa gave the scientific evidence of its use as anti-acne in 1973, who clinically examined the wax on patients suffering from acne vulgaris. The results revealed that jojoba oil could be used successfully to treat these conditions [10,13]. Its properties as a liquid wax allow the dissolution of sebum deposits within hair follicles due to an ability to penetrate the follicles and remove the comedome, thus clearing the skin.

Mosovich studied the effect of jojoba wax in the treatment of both acne vulgaris and psoriasis. He found that jojoba has high effectiveness in acne, with no secondary effects noted and no reports made of burning or itching. This efficacy indicates that jojoba oil may be used alone or in addition to other treatments. The antipsoriasis activity of jojoba oil is related to the positive keratoplastic and the slight keratolytic effect required to treat excessive scaling of the skin. Therefore, jojoba oil may be used as an additional treatment [6]. 

#### 6.2.3. Anti-Inflammatory, Antipyretic, and Analgesic Activities

Possible anti-inflammatory effects of jojoba oil were investigated against both acute and chronic inflammation of the skin. The possibility that jojoba oil could be beneficial in treating pain and reducing edema resulting from thermal and sunburns was demonstrated [10]. Habashy et al. conducted a study in 2005 that demonstrated this reduction of edema and prostaglandin E2 content in rats, further supporting this potential of jojoba. The anti-inflammatory effect of jojoba oil involved the blockage of both cyclooxygenase II and lipoxygenase enzymes [7,26]. 

This work is confirmed by a controlled clinical trial evaluating the short-term effectiveness of jojoba liquid wax as local treatment of Napkin rash. The results were compared to standard treatment of a combination of triamcinolone acetonide, nystatin, neomycin, and gramicidin. Jojoba liquid wax was found as effective as the use of that combination in the treatment of Napkin rash. However, jojoba had the advantage of being safer due to the lack of systemic side effects that are usually present using the combination mentioned above [10].

#### 6.2.4. Antimicrobial Activity

Jojoba oil has been shown to have an intense inhibitory action on the growth of *Tubercle bacilli*, *leprosy bacilli*, and *Brucelli* [11]. The liquid wax could help dissolve the solid wax coatings around the bacilli due to the chemical structure similarity between jojoba waxes and the fats forming the sheath of the bacilli, which prevents the penetration of the antibiotics. A combination of antibiotics and jojoba oil as penetrating oil may be effective in treating those serious diseases [6,46]. Furthermore, alcoholic extracts of the jojoba root have demonstrated antimicrobial activity against several pathogens, including *Bacillus cereus*, *Salmonella typhimurium*, and *Candida albicans*. This activity is attributed to the alkaloid, saponin, and steroid content of the root extract.

#### 6.2.5. Other Activities

Jojoba oil has also demonstrated a beneficial effect against hyperglycemia-induced oxidative stress. Cyanogenic glycosides and other components found in the seed extract markedly decreased ROS and caspase-3 activation and improved antioxidant defense, inhibiting p22phox and increasing nuclear factors—this activity may serve as a useful tool for combating diabetes [46,47]. Manoharan et al. support this theory, having conducted a report in 2016 that discussed the potential of jojoba oil to inhibit or manage diabetes due to its antioxidant properties. Jojobenoic acid present in the alcoholic seed extract has also demonstrated protection against FB1-induced hepatotoxicity in rat liver. Similar results were confirmed in a recent study that indicated jojoba seeds induced a decrease in body weight, fat mass, insulin resistance, oxidative stress, liver steatosis and renal complications. The results demonstrated the beneficial effect of jojoba against metabolic syndrome and oxidative stress [48].

Clarke and Yermanos were the first to study the lowering effect of jojoba oil on the serum cholesterol by their studies on the blood cholesterol level in rabbits where blood cholesterol level was reduced 40% in rabbits given 2% jojoba oil and 1% cholesterol in the diet for 30 days compared to the result obtained when 1% cholesterol is given alone [49].

The concomitant administration of jojoba oil with fipronil ameliorated the toxic effects of fipronil on liver, brain, and kidney with improvement of the antioxidant status, the rate of apoptosis, and the histopathological alterations. This positive effects were evidenced by the combating effect on the oxidative stress in liver, brain, and kidney as indicated by lowering the malondialdehyde (MDA) and nitric oxide (NO) levels with elevating in glutathione (GSH) level and activities of superoxide dismutase (SOD) and catalase (CAT). In addition, there is a marked lowering of the elevated serum levels of hepatic markers alanine aminotransferase (ALT), aspartate transaminase (AST), alkaline phosphatase (ALP), and lactate dehydrogenase (LDH) and the γ-aminobutyric acid (GABA) level in the brain [50].

Biorefinery processes can be employed to produce jojobyl alcohols from jojoba oil (11-eicosenol, 13-docosenol, and 15-tetracosenol), which have been explored as antivirals. Jojoba oil also holds value in the industry as anti-rodent treatment, insecticides, and bioenergy production. Furthermore, the presence of compounds with cyclooxygenase-2 (COX-2) inhibitive properties in jojoba leaf extracts holds potential in anticancer treatments. COX-2 inhibitors lead to an increase in the rate of apoptosis and a decrease in the invasiveness of cancer cells alongside angiogenesis reduction.

## 7. Pharmaceutical Uses

It is preferred to formulate it into a pharmaceutical preparation to achieve a maximum benefit of any biologically active natural compound. Owing to jojoba’s high skin moisturizing ability, high resistance to oxidation, its ability to penetrate the skin, and its ability to solubilize insoluble drugs, jojoba has been investigated as an excipient in different dosage forms. In an attempt to compile most of the work done on jojoba oil either as an active pharmaceutical ingredient or as an excipient, all the efforts published in PubMed have been collected, and most of the relevant information is reported in the following section. It is summarized in Table 7. 

### 7.1. Topical Preparations

Jojoba was incorporated in topical preparations to enhance the efficacy of drugs used to treat skin diseases [1]. A previous study demonstrated the ability of jojoba oil to solubilize lycopene, which is an important antioxidant with low solubility in both water and oil. The improved solubility enables the formulation of lycopene into liquid and transparent products for pharmaceutical uses [2]. Shevachman et al. reported the successful preparation of microemulsion using jojoba wax as the oily phase using different surfactants and cosurfactants, in which the content of jojoba oil determined the transition from the water-in-oil to bicontinuous and to oil-in-water structures [3]. 

Diclofenac sub-micron emulsion formulated using 20% jojoba oil showed an enhanced anti-inflammatory effect compared to marketed Voltaren^®^ Emulgel^®^ cream, which was attributed to jojoba penetrative properties [4]. Thakur et al. reported the preparation of jojoba oil-based emulsion of benzoyl peroxide for the treatment of acne. Based on jojoba oil emollient effect, anti-inflammatory, and antibacterial properties, the study resulted in a significant reduction in skin irritation and dryness caused by benzoyl peroxide. It enhanced its therapeutic effect [5].

Methotrexate-loaded jojoba oil-based microemulsion was proved to be clinically safe and effective in treating psoriasis vulgaris due to its moisturizing and anti-inflammatory effects [6]. Another jojoba oil-based microemulsion loading the synthetic retinoid tazarotene revealed a better therapeutic effect in psoriatic patients than the marketed product with no irritation and a double increase in tazarotene skin deposition [7].

Stable valacyclovir solid lipid nanoparticles were successfully prepared using jojoba oil. The prepared nanoparticles were stable with high entrapment efficiency of valacyclovir, which can be an effective delivery system in treating viral infections in humans [8]. In addition, nanostructured lipid carriers (NLC) were developed in semisolid preparation using jojoba oil as the liquid lipid. The in vivo studies showed a great increase in skin hydration and reduction of transepidermal water loss, which may result in the improvement in the symptoms of some skin disorders such as eczema [9].

Previous studies demonstrated the successful use of jojoba oil as an excipient for different topical antifungal preparations. Shahin et al. revealed the high physical stability of clotrimazole-loaded jojoba oil-based emulgels with a superior antimycotic activity against Candida albicans compared to the commercially available formulation Candistan^®^ and Canesten^®^ [10,11]. In addition, fluconazole dissolved in jojoba oil in the oily phase of microemulsion gel showed superior antifungal activity against Candida albicans with the widest zone of inhibition in comparison to fluconazole solution [12]. Moreover, El-Hadidy et al. explored the use of jojoba oil to formulate the poorly water-soluble voriconazole in microemulsion (ME) for topical application. Jojoba oil-based MEs showed good physical and rheological properties upon storage for 12 months at ambient conditions, and in vitro permeation studies revealed that they were able to sustain voriconazole release up to 42 h. In addition, voriconazole-loaded MEs showed significantly better antifungal activity against *Candida albicans* than the drug solution [13].

### 7.2. Cosmetic Products

Jojoba oil was reported for its use as a conditioning agent due to its emollient properties. The addition of jojoba oil to thioglycolate-based straightening emulsions benefited the hair fiber, allowing a little protein loss, protection to hair thread, and improved the breakage resistance [14]. In an attempt to benefit from the jojoba oil chemical backbone, Touitou and Godin formulated skin non-penetrating sunscreens (NPSUN) as new photo protectors from UV harmful radiation. The idea depends on the conjugation of jojoba oil with UV sunscreen molecules as methoxycinnamate to form new filters. The formulated NPSUNs exhibited high skin substantivity, decreasing the need for a frequent application with no in vitro permeation of methoxycinnamate-NPSUN across the skin for 24 h [15,16].

### 7.3. Transdermal and Intradermal Preparations

Jojoba oil has been investigated as a penetration enhancer in the fabrication of transdermal patches to deliver olanzapine. The fabricated patch was stable with good physical properties and increased drug skin flux [17]. Intradermally administrated Bacillus Calmette–Guérin (BCG) vaccine has been successfully encapsulated in small-sized agarose microcapsules and small-diameter alginate beads prepared by the emulsification of the hydrogel within jojoba oil. The freeze-drying microcapsules were stable for 12 months of storage at room temperature [18,19].

### 7.4. Parenteral Preparations

Novel nanocapsules for parenteral administration were prepared using vegetable oils such mango, jojoba, pequi, oat, annatto, calendula, and chamomile as the lipid core instead of the capric/caprylic triglyceride. All the formulated nanosystems were compatible regardless of the oil type; however, nanocapsules formulated using jojoba were the most favorable due to their optimum particle size characteristics [20].

### 7.5. Inhalable Preparations

The dry nanoemulsion powder prepared by jojoba oil had a good particle size distribution and an improved mass median aerodynamic diameter. The nanoemulsion showed higher anti-inflammatory effect on LPS-induced acute lung injury compared to than dexamethasone with a decrease in total protein content and downregulation of tumor necrosis factor-alpha (TNF-α), interleukins-1beta/6 (IL-1β/6), and NF-κB p65. In addition, it showed higher anti-inflammatory and anti-oxidation effect on H2O2-induced acute lung injury through the elimination of reactive oxygen species (ROS), increasing of superoxide dismutase (SOD), decreasing in of lipid peroxide malondialdehyde (MDA) and glutathione (GSH), and inhibition of caspase-3 expression [72].

### 7.6. Anticancer Preparations

Jojoba oil was incorporated in anticancer drug delivery systems. Solid nanoemulsion containing a combination of imiquimod, a Toll-like receptor 7 (TLR7) agonist, and model peptide antigen SIINFEKL for the topical treatment of different types of precancerous skin lesions and skin cancer, as a new perspective to avoid invasive technique and enhance dermal antigen administration [21]. Flores-Villaseñor et al. reported on the formulation of physically stable biocompatible o/w microemulsions with Jojoba oil as the oily phase for the delivery of paclitaxel. The microemulsion could load paclitaxel in a concentration of 0.3 mg/mL, which provides a new alternative nanodevice for cancer treatment [22]. In another study, authors were able to prepare positively charged micelles from bolaamphiphiles synthesized using natural jojoba oil. The prepared micelles delivered small interfering RNAs (siRNAs) to cancer cells with relatively low toxicities in vitro and in vivo and protected the nucleic acid from degradation [23].

## 8. Industrial Applications 

### 8.1. Synthesis Polyurethanes Polymers 

Jojoba oil has a wide range of industrial applications especially in polymers synthesis. Polymers are versatile materials that are used in a wide range of industries. Polyurethanes (Pus) are commonly used synthetic polymers involved in a wide range of applications, including furniture, automobiles, clothes, shoes, elastomers, coatings, and insulation. The usefulness of polyurethane is related to its excellent properties, including outstanding mechanical strength, strong chemical resistance, and light weight [73].

The most popular source of polyols used in the production of PUs is petrol; however, the researchers were forced to substitute petrol with clean natural resources such as vegetable oils, especially seed oils, due to the need for saving the environment. Seed oils are biodegradable, abundant, inexpensive substituents that entice many researchers to use them to produce PUs. This process converts different seed oils into reactive polyols by introducing hydroxyl groups into their structure with the consequent production of PUs with varying mechanical and thermal properties [74].

Jojoba oil has been used to produce PUs after functionalizing the oil by mercaptoethanol in a single step reaction to yield a diol with two hydroxyl functions, followed by catalyst-free reaction with different isocyanates to obtain different polyurethane prepolymers. Then, the chain extenders were added to obtain linear and cross-linked polyurethane materials characterized by exhibit good flexibility and high thermal stability [75]. The same group makes a major change through a high reactivity synthesis of cyclic carbonate monomers to generate biobased PolyHydroxyUrethane (PHU) from jojoba and castor oils. The first step involved thiol-ene coupling with thioglycolic acid to functionalize jojoba oil, followed by esterification with glycerol carbonate to yield new dicyclic carbonates. By aminolysis with different diamines, the dicyclic carbonates were used to synthesize linear PHUs. The cyclic carbonates produced are recommended due to the absence of solvents and catalysts during the manufacturing process and the production at low temperatures and thus with good reactivity [76].

More recent research has extended the development of linear PUs from synthesized diol Jojoba with different diisocyanates as a catalyst-free polycondensation reaction. PUS was versatile depending on the nature of the disocyanate used, with good thermal instability and regulated characteristics. Bio-based PUs are characterized by their improved solubility, allowing their casting with cellulose nanocrystals or cellulose nanofibrils to produce strong nanocomposites [77].

### 8.2. Other Industrial Uses

A rubber-like material is obtained from sulfurized jojoba oil, which is applied in linoleum manufacturing and ink printing composition, the paint and varnish industries, and the chewing gum industry [78]. One of the most extensive applications of jojoba oil includes an extreme temperature/extreme pressure lubricant in the form of sulfurized oil, which can bear high temperature and pressure without changing its viscosity [6,12,13,79]. Its stability at elevated temperatures permits the constant provision of a thin-film lubricating border, which is of remarkable necessity in decreasing frictional wear and temperature increase, which helps directly to extend the life span of the lubricating oil and indirectly to protect the automobile parts [13]. The study of the utilization of jojoba oil as a lubricant for some of the petroleum-derived products proved its improvement of certain desirable characteristics as antirust, antifoam, anti-wear, and friction reduction properties [80,81]. 

Nasser et al. explored the application of jojoba polymers as a lubricant and evaluated its viscosity index and pour point depressants when compared to homopolymer. The results showed that the viscosity index increases with increasing the alkyl chain length of both α- olefins and acrylate monomers. The pour point improved for additives based on alkyl acrylate. [82].

Another industrial application is its use as a surfactant due to the long alkenyl (jojobenyl, erucyl) alcohols obtained by hydrolysis, as its combination with polyethylene glycol chains customizes surfactants with desired hydrophile–lipophile balance [13].

The synthesis of methyl esters from jojoba oil allows its commercial application for the production of biodiesel. The results explored the promising future of jojoba oil as an oil feedstock for cultivation in comparatively dry areas [83,84]. The use of ultrasound technology was successfully applied to reduce the reaction time and temperature and increase biodiesel yield by reducing the cost and energy, contributing to a cleaner, safer and green technology for biodiesel production [84]. In addition, Jojoba oil is regarded as an excellent renewable feedstock to produce replacements for petroleum-derived transportation fuels and chemicals [85]. A recent study showed that the blend of jojoba oil with diesel fuel leads to a clear reduction in NOx and hydrocarbon (HC) emissions but indirectly impacts CO emission due to its high viscosity. Moreover, jojoba oil in the blends adversely influences thermal radiation to furnace walls due to the less sooting tendency of the flame when jojoba is present [86].

Moreover, jojoba oil has been used in the leather industry as a fat-liquoring agent, which verified significant improvement in the mechanical properties of leather, such as tensile strength and elongation at break [5,87]. 

The application of jojoba oil as an alternative collector for the selective separation of apatite and calcite minerals showed high selectivity between calcite and apatite, improving their selective flotation by using jojoba oil at a slightly acid medium, without the use of depressants [88].

Jojoba oil has been implemented with castor oil for the synthesis of PolyHydroxyUrethane polymer [76]. Other industrial uses include extraction and separation of isotopes such as Uranium (VI), Thorium (IV), and Plutonium (IV); and antifoaming agents in isolation of penicillin and tetracycline [9]. Jojoba oil replaced sperm whale oil as lamp oil, and it is used as solid wax to mix with and improve paraffin candies, microporous polyethylene film from microencapsulation of oil, and fatty acid amides as a lubricant for polyethylene film extrusion [13].

## 9. Toxicity of Jojoba Oil 

No acute toxicity was found when crude jojoba oil was fed to mice; i.e., the LD_50_ is more than 160 g/Kg. In ocular irritation tests on rabbits, jojoba oil refined by deodorization or deodorization and discolorations caused some reversible conjunctival irritation 1 h after oil application. The reverse action was completely cleared 24 h after ocular application. In 15- and 30-day-old guinea pigs, patch tests did not cause pathological inflammation. A light swelling of the epidermis occurred 30 days after topical application, which was less pronounced than that induced by liquid paraffin and more than that induced by olive oil. However, the effects on the animal skin were reversible and may have been caused by the occlusive nature of the oil film. In addition, prolonged daily subcutaneously injections in rats did not result in any histopathological changes of blood or urine analyses. Only a mild local reversible granulomatous reaction in the injected area indicated that jojoba oil is slightly irritant. Patch tests on humans did not reveal allergic reactions except in hyperallergic people. Prick tests with people exposed earlier to jojoba oil for two years revealed no allergic reactions to either the crude or refined oil.

A mixture of jojoba oil and hydrogenated jojoba wax was not mutagenic both with and without activation in the Ames assay [8,10,44,89]. Taguchi measured the safety of Jobacohol, which was produced by sodium reduction and molecular distillation. No acute toxicity was found in mice, and eye irritation was quite low. Repeated patch tests in rabbits, where Jobacohol was compared with oleyl alcohol with both dissolved in jojoba oil, showed no difference between these materials. Irritation was quite low, and it was concluded that Jobacohol is quite safe. Jobacohol also showed no irritation in primary skin irritation tests in marmots and no sensitization in maximization-of-sensitization tests. Negative results were also obtained in mutagenicity tests with *Salmonella typhimurium* and *Escherichia coli* [6].

Tests of primary irritation on humans with either healthy skin or humans suffering from contact dermatitis were also carried out. The results revealed no skin irritation concerning Jobacohol or oleyl alcohol. Phototoxicity tests on humans also showed that Jobacohol was quite free of this effect [6].

## 10. Conclusions

In this review, we shed light on one of the most economically important crops, jojoba. Although limited phytochemical work has previously been conducted on the different plant extracts, the composition of the liquid wax obtained by direct expression of the seeds has been thoroughly investigated. The plant appears to be a source of golden oil, which shows high structural similarity to spermaceti wax, involved in many pharmaceutical products. Traditionally, it is used for many skin and scalp disorders. The seed cake is used safely as a food and has many applications in the food industry due to its high fat and protein content. Most of the previously conducted biological work has been directed to prove the claimed emollient effect and then further extended to evaluate the oil’s anti-inflammatory, analgesic, and antipyretic properties. In addition, the plant has shown substantial activity as an antibacterial and antiviral agent. Interestingly, the plant extract shows a promising antidiabetic and antihypercholesterolemia. In conclusion, the jojoba tree represents an attractive source for the future development of new medication that could be identified and characterized using the new tools available in biochemical, physicochemical, and biological domains.

## Figures and Tables

**Table 1:** Chemical structures for the most abundant wax ester components in jojoba wax.

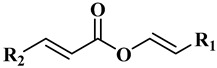	
R_1_ = C_20_H_41_, R_2_ = C_17_ H_35_	Docosenyl eicosenoate (**1**)
R_1_ = C_18_H_37_, R_2_ = C_17_ H_35_	Eicosenyl eicosenoate (**2**)
R_1_ = C_18_H_37_, R_2_ = C_19_ H_39_	Eicosenyl docosenoate (**3**)
R_1_ = C_16_H_33_, R_2_ = C_19_ H_39_	Docosenyl docosenoate (**4**)
R_1_ = C_18_H_37_, R_2_ = C_17_ H_33_ (C9)	Eicosenyl oleate (**5**)
R_1_ = C_20_H_41_, R_2_ = C_17_ H_33_ (C9)	Docosenyl oleate (**6**)

**Table 2:** The composition of free fatty alcohols and fatty acids derived from jojoba oil.

Alcohols	(%)	Acids	(%)
Tetradecanol	trace	Dodecanoic	trace
Hexadecanol	0.1	Tetradecanoic	trace
Heptadec-8-enol	trace	Pentadecanoic	trace
Octadecanol	0.2	Hexadecanoic	1.2
Octadec-9-enol	0.7	Hexadec-7-onoic	0.1
Octadec-11-enol	0.4	Hexadec-9-enoic	0.2
Eicosanol	trace	Heptadecenoic	trace
Eicos-11-enol	43.8	Octadecanoic	0.1
Hecos-12-enol	trace	Octadec-9-enoic	10.1
Docosanol	1.0	Octadec-11-enoic	1.1
Docos-12-enol	44.9	Octadecadienoic	0.1
Tetracos-15-enol	8.9	Octadecatrienoic	trace
Hexacosenol	trace	Nonadecenoic	trace
		Eicosanoic	0.1
		Eicos-l1-enoic	71.3
		Eicosadienoic	trace
		Docosanoic	0.2
		Docos-13-enoic	13.6
		Tricosenoic	trace
		Tetracosenoic	trace
		Tetracos-15-enoic	1.3

**Table 3:** The average percentage of sterols content in jojoba oil.

Sterol	Sterol Fraction (%)	Total Wax (mg/kg Seed)
Unidentified	0.4	16
Stigmasta-5,25-dien-3β-ol	0.6	24
Fucosterol	0.6	24
Isofucosterol	4.1	163
Cholesterol	0.8	32
Stigmasterol	6.7	266
Campesterol	16.9	672
Sitosterol	69.9	2780

**Table 4:** Chemical structures for the most abundant flavonoids in jojoba.

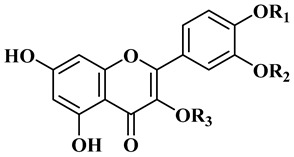			
	R1	R2	R3
Quercetin (**12**)	H	H	H
Isorhamnetin (**13**)	H	Me	H
Quercetin 3-methyl ether (**14**)	H	H	Me
Quercetin 3,3′-methyl ether (**15**)	H	Me	Me
isorhamnetin 3-*O*-glucoside (**16**)	H	Me	Glc
Quercetin-3-*O*-glucoside (**17**)	H	H	Glc
Typhaneoside (**18**)	Me	H	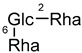
Isorhamnetin 3-*O*-rutinoside (**19**)	Me	H	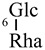
Quercetin 3-*O*-rutinoside (**20**)	H	H	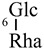

**Table 5:** Solubility characteristics of jojoba oil in common organic solvents at 15 °C ^a^.

Solvent	mL of Solvent	Observation ^b^
Water	5.0	I
	10.0	I
Acetic acid	10.0	I
	40.0	I
	50.0	S
Methanol	1.0	I
	10.0	I
	40.0	S
Ethanol	1.0	I
	5.0	I
	20.0	S
t-Amyl Alcohol	1.0	S
1-Butanol	1.0	S
Acetone	1.0	I
	3.0	I
	8.0	I
Benzene	1.0	I
Toulene	1.0	I
Carbon Tetrachloride	1.0	I
s-Tetrachlocthane	1.0	I
Diethylether	1.0	I
Tetrahydrofuran	1.0	I
Hexane	1.0	I
Cyclohexane	1.0	I
Dimethylformamide	1.0	I
	10.0	I
	30.0	S
Dimethylsulfoxide	1.0	I
	5.0	I
	20.0	S
Acetonitrile	1.0	I
	10.0	I
	30.0	S
Aniline	2.0	S
m-Cresol	2.0	S

^a^ all measurement used 0.2 g ^b^ I = insoluble; S = soluble.

**Table 6:** Some physical properties of jojoba oil as reported in the literature [31].

Freezing point, °C	10.6–7.0
Melting point, °C	6.8–7.0
Boiling point at 757 mm under N2, °C	389
Heat of fusion by DSC, Cal/g	21
Refractive index at 25 °C	1.465
Dielectric constant, 27 °C	2.680
Specific conductivity, 27 °C, mho/cm	8.86.10–13
Specific gravity, 25/25 °C	0.863
MV-1 rotor in MY cup, cp	35
Plate and cone with PK-1, cp	33
Brookfield, spindle #1, 25 °C, cp	37
Cannon–Fenske, 25 °C, cp	50
Cannon–Fenske, 100 °C centistokes	27
Smoke point, °C	195
Flash point, °C	295
Fire point, °C	338
Iodine value	82
Saponification value	92
Acid value	<2
Acetyl value	2
Unsaponifiable matter, %	51
Total acids, %	52
Iodine value of alcohols	77
Iodine value of acids	<76
Average molecular weight of wax esters	606

**Table 7:** A summary of pharmaceutical dosage forms containing jojoba oil.

Dosage Form	Drug	Ingredients	Use/effect of Jojoba Oil	Ref.
Microemulsion	Antioxidant lycopene	Jojoba oil, alcohols, nonionic surfactant (Brij 96V)	To solubilize lycopene	[51]
Microemulsion	-	Jojoba oil, alcohols, different nonionic surfactants, namely Brij 96V and Tweens, and water	To study the effect of Jojoba oil content on the type of the microemulsion	[52]
Sub-micron emulsion	Diclofenac (Diethyl ammonium)	Jojoba oil, purified egg lecithin, Cremophor EL surfactant, and water	To increase the anti-inflammatory effect of topical preparations of diclofenac	[53]
Gellified emulsion	Anti-acne agent, Benzoyl peroxide	Lipophilic surfactant (Span 60), jojoba oil, hydrophilic surfactant (Tween 20), propylene glycol, methyl paraben, propyl paraben, disodium EDTA, butylated hydroxy toluene, Carbopol 940, and water	To decrease the skin irritation and dryness caused by benzoyl peroxide	[54]
Microemulsion	Methotrexate	Jojoba oil, Tween 80, Span-85 and water	Treatment of psoriasis vulgaris.	[55]
Microemulsion	Synthetic retinoid tazarotene	Jojoba wax, labrasol/plurol isostearique and water	Treatment of psoriasis and increase in skin deposition of tazarotene	[56]
Solid lipid nanoparticles	Valacyclovir hydrochloride	Glyceryl monostearate. jojoba oil, polyethylene Glycol 400, Tween 80, and water	To benefit from jojoba oil moisturizing and stabilizing activity in the treatment of viral infections in humans	[57]
Nanostructured lipid carriers	-	Glyceryl behenate, jojoba oil, Tween 80, cetrimide, glycerine, Carbopol 934 or Carbopol 980, triethanolamine, and water	To improve symptoms of some skin disorders like eczema	[58]
Emulgels	Clotrimazole	Jojoba oil, hydroxypropyl methylcellulose (HPMC) Carbopol 934, Span 60, Brij 35, triethanolamine, propylene glycol, and water	An excipient for different topical antifungal preparations	[59]
		Hydrophobically modified co-polymers of acrylic acid, namely Pemulen TR1 and TR2, jojoba oil, and water		[60]
Cutina lipogels	Fluconazole	Cutina, Jojoba oil	An excipient for fluconazole topical drug delivery	[61]
Microemulsion gel		Jojoba oil, Brij 96, Capmul and, water		
Straightening emulsions	-	Jojoba oil, ammonium thioglycolate, self-emulsifying wax, oleth-3, mineral oil, propylene glycol, aqua, and preservative blend	As a conditioning agent added to the emulsion	[62]
Skin non-penetrating sunscreens	-	Jojoba oil, methoxycinnamate	To link UV sunscreen molecules as methoxycinnamate to jojoba oil to form new filters	[63,64]
Transdermal patch	Olanzapine	Jojoba oil, Eudragit polymer	As a penetration enhancer in transdermal delivery	[65]
Small-sized agarose microcapsules	Bacillus Calmette–Guérin (BCG) vaccine	Agarose, jojoba oil	An excipient	[66,67]
Small-diameter alginate beads		Calcium alginate matrix, jojoba oil		
Nanocapsules		Jojoba oil, Poly(€-caprolactone) Tween 80, and Span 60	To study physical stability and the hemocompatibility of jojoba oil-based nanocapsules for parenteral administration	[68]
Solid nanoemulsion	Imiquimod, a Toll-like receptor 7 (TLR7) agonist + SIINFEKL antigen	Jojoba oil, sucrose fatty ester S-1670 and water	An excipient	[69]
O/W microemulsions	Paclitaxel	Jojoba oil, d-α-tocopherol polyethylene glycol 1000 succinate (TPGS-1000), isobutanol, and water	As an excipient to load paclitaxel for cancer treatment	[70]
Charged micelles	Small interfering RNAs (siRNAs)	Cationic lipids Bolaamphiphiles (GLH-58 and GLH-60) synthesized from jojoba oil	Starting material for the synthesis of lipids	[71]

## Data Availability

The data presented in this study are available on request from the corresponding author.
